# Predictive value of stress hyperglycemia ratio for perioperative hidden blood loss in patients with hip fractures: a retrospective cohort study

**DOI:** 10.3389/fmed.2025.1678269

**Published:** 2025-12-05

**Authors:** Yunyang Jia, Yan Huo, Minghui Yang, Ting Li

**Affiliations:** Department of Orthopedic Surgery, Beijing Jishuitan Hospital, Capital Medical University, Beijing, China

**Keywords:** stress hyperglycemia ratio, hip fracture, hidden blood loss, SHR, HBL

## Abstract

**Objective:**

To investigate the predictive value of stress hyperglycemia ratio (SHR) for perioperative hidden blood loss (HBL) in patients with hip fractures.

**Methods:**

A retrospective analysis was conducted on 152 patients with hip fractures admitted to our hospital from January 2021 to December 2023. Patients were divided into an HBL group (*n* = 52) and a non-HBL group (*n* = 100) based on the occurrence of HBL during the perioperative period. Univariate and binary Logistics regression analyses were used to identify influencing factors. The receiver operating characteristic (ROC) curve was employed to evaluate the predictive value of SHR for perioperative HBL in hip fracture patients.

**Results:**

There were statistically significant differences (*p* < 0.05) in terms of BMI, osteoporosis, SHR, surgical time, and postoperative drainage between the two groups. Binary Logistics regression analysis indicated that surgical time, postoperative drainage, and SHR were independent influencing factors for perioperative HBL in hip fracture patients (*p* < 0.05). The ROC analysis revealed an area under the curve (AUC) of 0.911, with a standard error of 0.024 (95% CI, 0.865 ~ 0.958) and a Youden index of 0.65. At this threshold, the sensitivity was 71.22%, and the specificity was 94.00%. Patients with SHR < 1.18 had higher EQ-5D scores, Hct, Hb, PLT, and RBC compared to those with SHR ≥ 1.18. Additionally, complications, length of hospital stay, readmission rates, CRP, APTT, and PT were lower in the SHR < 1.18 group, with statistically significant differences (*p* < 0.05).

**Conclusion:**

SHR demonstrates a certain predictive value for perioperative HBL in patients with hip fractures, suggesting its inclusion in evaluation models.

## 1 Introduction

In recent years, with the exacerbation of global population aging, the incidence of lower limb fractures in the older adult has shown a significant upward trend. Statistics show that in China, over 3 million older adult patients are hospitalized for lower limb fractures annually, with more than 50% of them requiring surgical treatment (1). Hip fracture, as a common and severe type of lower limb fracture, requires crucial perioperative management for patient prognosis, with hidden blood loss (HBL) during the perioperative period having a significant impact on outcomes (2). HBL refers to blood loss that occurs during and after surgery but is not directly observed (3). However, related researches in this area are still limited. The concept of stress hyperglycemia ratio (SHR) compares fasting blood glucose levels with glycated hemoglobin levels upon patient admission, providing a more accurate reflection of the patient’s metabolic stress status and overcoming the limitations of single blood sugar indicators. In recent years, SHR has demonstrated significant clinical value in predicting in-hospital mortality, disease severity, and long-term adverse risks (such as cardiovascular events and readmission risk) for patients (4, 5). Nevertheless, research on the application of SHR in predicting HBL during the perioperative period in hip fracture patients remains insufficient. Therefore, investigating the relationship between SHR and perioperative HBL in patients with hip fractures is of paramount importance for optimizing perioperative management, reducing transfusion requirements, and enhancing surgical safety. This study aims to evaluate the predictive value of SHR for perioperative HBL in hip fracture patients, with the goal of providing more precise blood loss prediction tools for clinical practices, guiding the formulation of perioperative transfusion strategies, and ultimately improving patient outcomes.

### 1.1 Research objects

One hundred fifty-two patients with hip fractures admitted to our hospital between January 2021 and December 2023 were retrospectively selected for this study. Patients were categorized into an HBL group (*n* = 52) and a non-HBL group (*n* = 100) based on the occurrence of HBL during the perioperative period, with the criteria set by Zong et al. (6): The HBL for all patients was strictly calculated based on the gross equation: HBL = total red cell loss - major red cell loss + blood transfusion volume. HBL ≥ 480 milliliters was defined as present and included in the blood loss group; conversely, HBL < 480 milliliters indicated no hidden blood loss and was included in the no blood loss group. The calculation method of total red blood cell loss and major red blood cell loss uses Nadler’s formula. The inclusion criteria were as follows: (1) Patients diagnosed clinically with hip fractures (7) and meeting surgical indications. (2) All surgeries were performed at our hospital. (3) Patients aged over 18 years. (4) Unilateral fractures. (5) Complete clinical data. The exclusion criteria were as follows: (1) Patients with concomitant pathological fractures or open fractures. (2) Patients with psychiatric disorders. (3) Pre-existing diabetes or other metabolic disorders, blood system disorders, etc. (4) Patients who received blood transfusion or blood product therapy before surgery.

## 2 Research methods

### 2.1 General data collection

Patient general information was collected using the electronic medical record system.

### 2.2 Calculation of SHR

SHR (8) == fasting blood glucose level at admission (mmol/L) / [(1.59 × glycated hemoglobin (HbA1c) %)−2.59].

### 2.3 EuroQol five dimensions score (EQ-5D)

EQ-5D scores (9) filled out by patients before discharge were collected to assess the patients’ health-related quality of life.

### 2.4 Statistical analysis

The experimental data collected were analyzed with SPSS 27.0 (International Business Machines Corporation, Armonk, New York, USA). The normality of the distribution in the experimental data was assessed using the Kolmogorov–Smirnov test. For metric data that conformed to a normal distribution, results were presented as X̄ ± S, and comparisons were made using independent sample t-tests. For data not following a normal distribution, the median (interquartile range, IQR) was represented as MQ2 (Q1, Q3), and calculations were performed using the Mann–Whitney U test. Count data were expressed as frequencies or rates, with comparisons conducted with *χ*^2^ test or Fisher’s exact test. Factors were analyzed with univariate and binary Logistics regression analysis. The predictive value of SHR for perioperative HBL in hip fracture patients was evaluated with the ROC curve. A significance level of *p* < 0.05 was considered statistically significant for differences.

## 3 Results

### 3.1 Univariate analysis of factors influencing perioperative HBL in hip fracture patients

Statistical significance was observed in the comparison of BMI, osteoporosis, SHR, surgical time, and postoperative drainage between the two groups of patients (*p* < 0.05), as shown in Table 1.

**Table 1:** Univariate analysis of factors influencing perioperative HBL in hip fracture patients.

Indicator		HBL group (*n* = 52)	Non-HBL group (*n* = 100)	*Z/t/χ*^2^ value	*p* value
Age (years)		82.50 (78.00, 87.50)	81.00 (78.00, 84.50)	−1.681	0.093
Gender	Male	25	48	—	0.993
	Female	27	52		
BMI (kg/m^2^)		21.53 ± 2.40	23.72 ± 1.49	−5.145	<0.001
Injury to surgery (days)		4.00 ± 0.94	3.87 ± 0.83	−0.806	0.420
Hypertension	Yes	40	66	1.934	0.164
	No	12	34		
Alcohol drinking history	Yes	20	36	0.089	0.765
	No	32	64		
Smoking history	Yes	25	43	0.357	0.550
	No	27	57		
Osteoporosis	Yes	30	30	10.981	0.001
	No	22	70		
SHR		1.41 (1.26, 1.54)	0.98 (0.90, 1.09)	−10.068	<0.001
Fracture type	Femoral neck	18	48	2.495	0.114
	Intertrochanteric	34	52		
Surgery time (min)		131.40 ± 19.08	95.42 ± 8.03	16.317	<0.001
Postoperative drainage	Yes	36	37	14.238	<0.001
	No	16	63		

BMI, body mass index; SHR, stress hyperglycemia ratio; Hct, hematocrit; Hb, hemoglobin; CRP, C-reactive protein; APTT, activated partial thromboplastin time; PT, prothrombin time; PLT, platelet count; RBC, red blood cell count; ASA, American Society of Anesthesiologists.

Age, SHR using the Mann–Whitney U test.

### 3.2 Binary logistics regression analysis of factors influencing perioperative HBL in hip fracture patients

Using variables that were significant in univariate analysis as independent variables, values were assigned to them and further analysis was conducted with HBL as the dependent variable (HBL = 1, non-HBL = 0). The results of the binary logistics regression analysis indicated that surgical time, postoperative drainage, and SHR were independent influencing factors for perioperative HBL in hip fracture patients (*p* < 0.05), as shown in Tables 2, 3.

**Table 2:** Variable assignment.

Influencing factor	Assignment
BMI	Original value
Osteoporosis	No = 0, Yes = 1
SHR	Original value
Surgical time	Original value
Postoperative drainage	No = 0, Yes = 1

**Table 3:** Binary logistic regression analysis of factors influencing perioperative HBL in hip fracture patients.

Factor	*β*	Standard error	Wald	P	Exp (*β*)	95% CI	
						Lower limit	Upper limit
BMI	0.759	0.742	1.046	0.306	2.136	0.499	9.143
Osteoporosis	−2.011	1.897	1.124	0.289	0.134	0.003	5.512
Surgical time	0.768	0.279	7.589	0.006	2.156	1.248	3.725
Postoperative drainage	−8.898	3.355	7.036	0.008	0.000	0.000	0.098
SHR	109.459	42.826	6.533	0.011	3.446	1.212	9.797
Constant	−96.672	42.936	5.069	0.024	0.000	—	—

### 3.3 ROC curve analysis of predictive value of indicators

**Figure 1:**
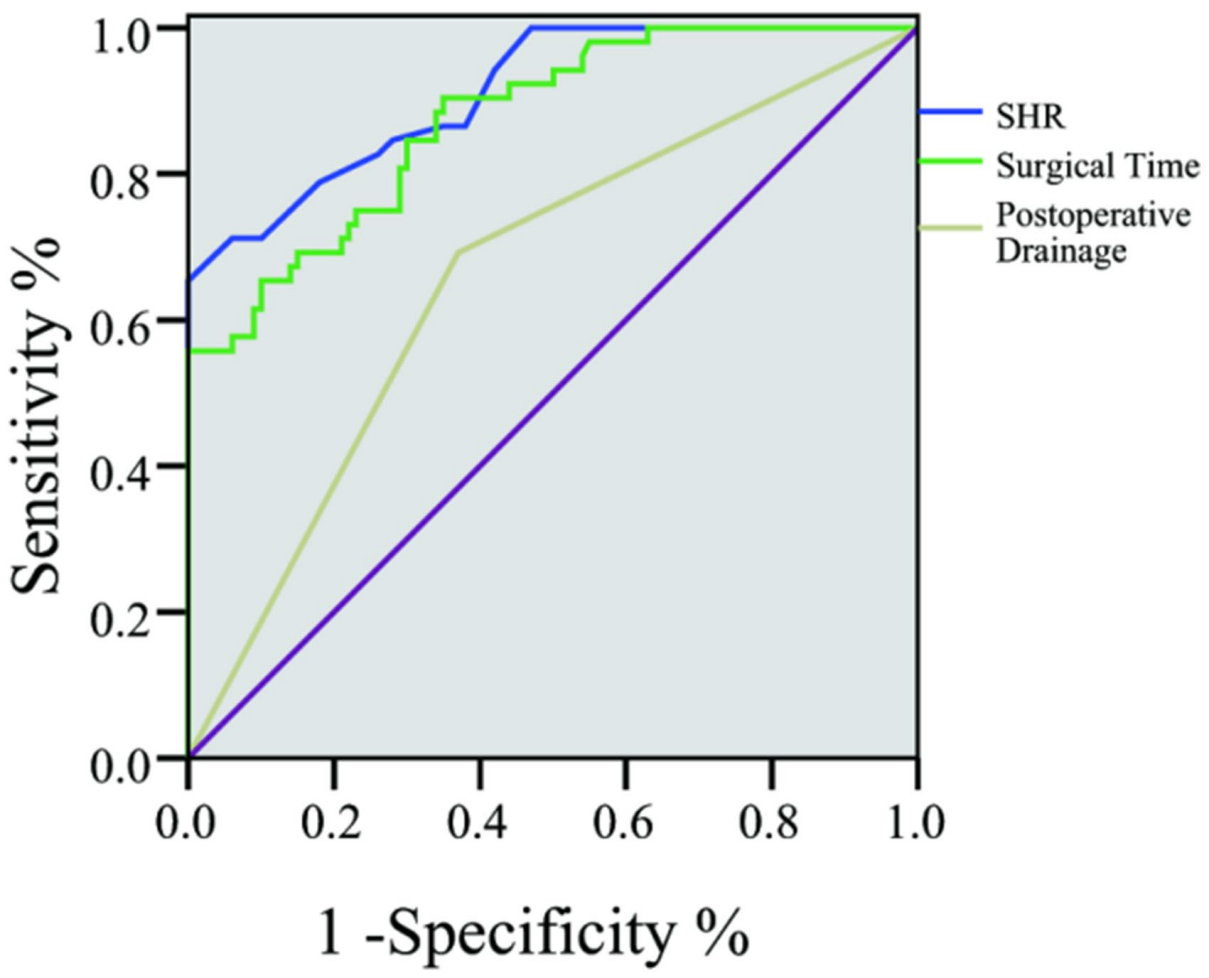
ROC curve.

The ROC analysis revealed that the area under the curve for SHR was 0.911, with a standard error of 0.024 (95% CI, 0.865 ~ 0.958) and a Youden index of 0.65. At this threshold, the sensitivity was 71.22%, and the specificity was 94.00%. See Table 4 and Figure 1 for details.

**Table 4:** ROC analysis results.

Indicator	AUC	Standard error	95% CI	Youden	Sensitivity	Specificity	Optimal cut-off value
Surgical time	0.874	0.029	0.818 ~ 0.931	0.56	90.44	65.21	109.5
Postoperative drainage	0.661	0.047	0.570 ~ 0.752	0.32	69.27	63.00	—
SHR	0.911	0.024	0.865 ~ 0.958	0.65	71.22	94.00	1.18

### 3.4 Optimal cut-off value of SHR and patient survival rate

Patients with SHR < 1.18 had higher EQ-5D scores compared to those with SHR ≥ 1.18, with statistically significant differences observed in the occurrence of complications, length of hospital stay, and readmission rate (*p* < 0.05). See Table 5 for details.

**Table 5:** Optimal cut-off value of SHR and patient survival rate.

Item		SHR < 1.18 (*n* = 100)	SHR ≥ 1.18 (*n* = 52)	*χ*^2^ value	*p* value
Complications	None	94	20	59.150	<0.001
	Pneumonia	0	4		
	Infection	2	11		
	Heart failure	0	4		
	Thrombosis	2	5		
	Delirium	2	9		
EQ-5D score (points)		80.97 ± 3.46	62.29 ± 10.55	16.154	<0.001
Length of hospital stay		4.50 ± 0.56	6.25 ± 0.93	14.460	<0.001
Readmission	Yes	1	8	12.708	<0.001
	No	99	44		

EQ-5D, EuroQol five dimensions score.

### 3.5 The optimal cut-off value of SHR and other patient laboratory parameters

Patients in the SHR < 1.18 group exhibited higher levels of Hematocrit (Hct), Hemoglobin (Hb), Platelet Count (PLT), and Red Blood Cell Count (RBC) compared to the SHR ≥ 1.18 group. Additionally, C-reactive Protein (CRP), Activated Partial Thromboplastin Time (APTT), and Prothrombin Time (PT) were lower in the SHR ≥ 1.18 group, with statistically significant differences observed (*p* < 0.05). See Table 6 for details.

**Table 6:** The optimal cut-off value of SHR and other patient laboratory parameters.

Item	SHR < 1.18 (*n* = 100)	SHR ≥ 1.18 (*n* = 52)	*χ*^2^ value	*p* value
Hct (%)	38.47 ± 1.30	32.96 ± 2.83	16.450	<0.001
Hb (g/L)	127.43 ± 5.07	109.67 ± 10.49	14.087	<0.001
CRP (mg/L)	6.83 ± 2.39	17.79 ± 5.89	16.248	<0.001
APTT (s)	32.26 ± 1.27	34.57 ± 2.18	12.238	<0.001
PT (s)	12.31 ± 0.44	13.53 ± 0.79	12.407	<0.001
PLT (10^9^/L)	254.63 ± 22.98	205.44 ± 23.59	11.935	<0.001
RBC (10^12^/L)	4.48 ± 0.27	3.90 ± 0.31	16.450	<0.001

## 4 Discussion

Through a retrospective analysis of clinical data from 152 hip fracture patients, this study found that SHR had significant value in predicting perioperative HBL. The results of the ROC curve analysis demonstrated that SHR exhibits high diagnostic efficiency. Setting the critical value of SHR at 1.18 enables accurate identification of most patients at risk of HBL, while reducing misdiagnosis rates.

Patients with hip fractures often experience a heightened state of stress during the perioperative period. Factors such as surgical trauma, anesthesia, and blood loss can trigger the secretion of significant amounts of insulin antagonistic hormones in the body, such as adrenal corticosteroids, cortisol, and catecholamines, leading to stress-induced hyperglycemia. This hyperglycemic state not only reflects the degree of the body’s stress response but may also increase the risk of HBL through mechanisms that affect coagulation function, vascular endothelial function, and more (10). For instance, elevated blood glucose levels can increase blood viscosity, slow down blood flow rates, prolong blood retention in tissue interstices, and promote the occurrence of HBL. The SHR combines two indicators, Fasting Plasma Glucose (FPG) and Hemoglobin A1c (HbA1c), to comprehensively assess a patient’s metabolic stress state. FPG primarily reflects blood glucose levels during acute stress, while HbA1c reflects average blood glucose levels over the past 2–3 months. Previous studies by Cannarsa et al. (11) have shown a relationship between SHR and bleeding, aligning with the viewpoint of this study. Therefore, SHR can distinguish between stress-induced hyperglycemia and chronic hyperglycemic states. In hip fracture patients, an elevated SHR often indicates a stronger metabolic stress response, which through various mechanisms, may promote the occurrence of HBL.

At the biological mechanism level, the potential pathways through which SHR predicts perioperative HBL in hip fracture patients involve several key processes. Firstly, concerning coagulation function, a hyperglycemic state significantly alters the physicochemical properties of blood. Elevated blood glucose concentrations increase blood viscosity and enhance platelet activity. These changes make blood more prone to forming microthrombi in the microcirculation (12). The formation of microthrombi not only blocks local blood vessels, affecting normal blood circulation, but also prolongs the retention time of blood in tissue interstices, increasing the risk of blood seepage into tissue interstices, ultimately leading to HBL. Secondly, oxidative stress triggered by high blood glucose is a crucial factor leading to vascular endothelial damage. In stress conditions, the body generates large amounts of reactive oxygen species (ROS) and other oxidative substances, which attack vascular endothelial cells, disrupting their structure and function, leading to a significant increase in vascular permeability. Damage to vascular endothelial cells weakens the barrier function of the vessel wall, making it easier for blood components to seep into tissue interstices, thereby promoting HBL occurrence (13, 14). Moreover, high blood glucose also activates the body’s inflammatory response, releasing various inflammatory cytokines such as tumor necrosis factor-alpha (TNF-α), interleukin-6 (IL-6), etc. These inflammatory factors not only exacerbate local tissue inflammation but may also promote HBL by affecting the integrity of the vessel wall. Inflammatory factors can loosen the connections between vascular endothelial cells, further increasing vascular permeability, making blood more prone to extravasation (15). In this study, patients with SHR ≥ 1.18 exhibited significantly higher levels of inflammatory and coagulation indicators such as CRP, APTT, and PT compared to the SHR < 1.18 group, further supporting the aforementioned biological mechanisms, indicating that the hyperglycemic state promotes HBL occurrence through multiple pathways involving coagulation function, vascular endothelial integrity, and inflammatory responses (16).

Additionally, this study also found that surgical time and postoperative drainage are independent factors influencing Hidden Blood Loss (HBL), and they may have complex interactions with SHR, collectively impacting the occurrence of HBL. Previous research by Hong et al. (17) has also indicated the relevance of surgical time to HBL following hip joint surgery, aligning with the findings of this study. Prolonged surgical time signifies increased surgical trauma and prolonged exposure of tissues to the external environment, which not only directly leads to tissue damage and increased bleeding but may also trigger a stronger stress response, leading to increased secretion of stress hormones, thereby elevating SHR and creating a vicious cycle that exacerbates the risk of HBL (18). Postoperative drainage, a routine procedure after surgery, aids in reducing local hematoma formation. However, it may also promote blood extravasation due to decreased tissue interstitial pressure. Simultaneously, the loss of drainage fluid can reduce effective circulating blood volume, leading to blood concentration, relative deficiencies in clotting factors, further affecting coagulation function, and indirectly promoting the occurrence of HBL. Previous research by Zong et al. (6) has also associated postoperative drainage with HBL postoperatively, in alignment with the findings of this study. Therefore, in clinical practices, for patients with longer surgical times or increased postoperative drainage, even if their SHR values are at a lower level, vigilance should be maintained regarding the risk of HBL occurrence. A comprehensive evaluation considering multiple factors is necessary to develop more precise perioperative management strategies, effectively reducing the adverse impact of HBL on patient outcomes.

The results of this study demonstrate that SHR exhibits high sensitivity and specificity in predicting perioperative HBL in hip fracture patients, suggesting its incorporation into assessment models. By calculating SHR, patients at risk of HBL can be identified early, enabling targeted preventive measures such as optimizing surgical plans, reducing surgical trauma, and enhancing postoperative monitoring. Individualized treatment plans can be developed based on the level of SHR. For instance, patients with higher SHR values can benefit from intensified blood glucose management to reduce stress responses and closely monitor HBL occurrences. SHR can also serve as a crucial indicator for evaluating patient prognosis. Patients in the SHR ≥ 1.18 group showed significantly higher rates of complications, longer hospital stays, and readmission rates compared to the SHR < 1.18 group, indicating a close relationship between SHR and patient outcomes.

While this study confirms the value of SHR in predicting perioperative HBL in hip fracture patients, certain limitations exist. Firstly, this study is retrospective and may introduce selection bias and information bias. Due to the limitations of previous data, there are problems such as failure to include other potential confounding factors. Secondly, the sample size is relatively small, which could impact result stability. Future researches can validate the predictive value of SHR further by expanding sample sizes and conducting prospective studies. Additionally, exploring the combined application of SHR with other biomarkers (such as inflammatory factors, coagulation indicators, etc.) could enhance prediction accuracy.

## 5 Conclusion

In summary, SHR has clinical value in predicting perioperative HBL in patients with hip fractures. SHR can comprehensively reflect a patient’s metabolic stress status, and can more accurately assess the patient’s acute stress response by combining the two indicators of FPG and HbA1c. This study found that the area under the prediction curve, as well as the sensitivity and specificity of SHR were high, indicating that it has extremely high diagnostic power in predicting HBL. Therefore, it is recommended to include SHR in the evaluation model of perioperative HBL in patients with hip fractures to guide clinical practice and improve patient prognosis.

## Data Availability

The original contributions presented in the study are included in the article/supplementary material, further inquiries can be directed to the corresponding author.
